# Cases of Small-Pox after Vaccination; Copied from the Minute Book of the Original Vaccine-Pock Institution, Broad-Street, Golden-Square

**Published:** 1812-08

**Authors:** 


*For the Medical and Physical Journal.*

*Cases of Small-pox after Vaccination; copied from the Minute Book of the original Vaccine-Pock Institution, Broad-street, Golden-square.*

WE have been permitted to transcribe the following most interesting minutes from the Record Book of the original Vaccine-Pock Institution—an Institution distinguished for its independence and courage in the assertion of truth during the violent contentions of other opposing parties.

MAY 5, 1812.—Mrs. Ancell, shopkeeper, No. 3, King-street, Five-fields, Chelsea, attended this day to state that she had three children ill of the small-pox after vaccination at this Institution, and a fourth ill of the natural small-pox, not previously vaccinated.

On consulting our registers, it appears that these three cases of asserted failure, had gone through the cow-pock at this Institution, viz.

1. Mary Anne Ancell, No. 401 on Dr. Nelson and Mr. Carpue's list, was vaccinated March 31, 1801, then eleven months old, at the breast, with eight-day matter, and went through the affection in a regular manner; with one fine large vesicle.

2. John Ancell, No. 1736 in Dr. Nelson and Mr. Doratt's list, was vaccinated Jan. 24, 1804, then fifteen months old, with eight-day matter, producing four distinct vesicles, and the usual satisfactory scabs.

3. Lucy Ancell, No. 2664 in Dr. Nelson and Mr. Doratt's list, vaccinated Sept. 3, 1805, with eight-day matter, then five months old; had six well characterised vesicles, viz. three on each arm, and two subsequent distinct scabs.

Friday, May 8.—Dr. Domeir and Dr. Pearson reported that they had visited the above patients last Wednesday, May 6. That they found one of the children, viz. John, had not more than thirty small black and brown scabbed eruptions, smaller than the small kinds of chicken-pock, and was in other respects pretty well. That Mary had a very numerous eruption, but distinct, and most of them were scabbed. Lucy had a still more numerous eruption; indeed, they were confluent on the arms and face; they were of the vesicular or bladdery kind, and in several parts bags of lymph hung from them; her face was swelled, and her eyes closed up for a day or two; but she is now walking about. George was exceedingly ill with the confluent sort; also bladdery, and more extensively confluent even than the former. The throat seemed a good deal affected, the respiration laborious, and the eyes almost closed up. The small-pox was in the neighbourhood at the time these children were taken ill; and many children had from time to time attended at the shop of this family when recovering from the small-pox, although they had not immediate intercourse with Mrs. Ancell's children.

It appears, upon the inquiry of the above physicians, 1. That Mary Anne, now aged twelve years, began to sicken twelve days ago, viz. last Friday week, April 24th; the eruption came out on Sunday the 26th. 2. John, now nine years of age, began to sicken the day following, viz. last Saturday but one, April 25th; the eruption came out on the Monday following. 3. Lucy, now aged seven years, sickened last Monday week, April 26th; and the eruption came out on Wednesday following. 4. George, aged four years, who had not been vaccinated, began to be ill last Tuesday week, April 27th; and the eruption came out on Thursday, April 30th.

Tuesday,

Tuesday, May 12.—Dr. Pearson reported, that he had again visited the children of Mrs. Ancell on Saturday evening last: Mr. Ewbank had also visited them on Friday. Mrs. Ancell had likewise attended at Dr. Pearson's lecture-room yesterday, with the three children, recovering from the small-pox after the cow-pock. He is sorry to report that the child, George, ill of the natural small-pox, died on Saturday morning, being the tenth day of the eruption.

Mrs. Ancell's three children, Mary, John, and Lucy, attended at the Institution this day; likewise Charlotte, another daughter, aged 17, who had the small-pox in the natural way severely when seven months old, and is now much scarred.

Mary seems to be nearly recovered, but has distinctly the marks upon her; the scabs having fallen off, it now appears that she has one very distinct scar upon the left arm from vaccination.

On John, very few marks from the small-pox can now be seen, he is so much recovered; three distinct scars on the left arm from vaccination are now seen.

From Lucy, the scabs are falling off very fast, but they are still very considerable and confluent on the lower arm. The cuticle is peeling off entirely from the fingers of both hands; three scars from vaccination are now perceivable upon the left arm, and one on the right.

The following remarks and conclusions are offered by Dr. Pearson:

1st. That in a certain proportion of cases, the constitution is not rendered unsusceptible of the small-pox, after going through the cow-pock in the most characteristic manner; for the symptoms and progress of the disease in the above cases were too distinctly marked to admit of a doubt of their being small-pox.

2dly. As a confirmation of what has happened in our practice and elsewhere, this is one of the most decisive examples of constitutional susceptibilities existing in certain families that has ever been observed.

3dly. Two of the cases were much more severe than have ever occurred at this Institution, and which, perhaps, have rarely happened in private practice after vaccination. The third was so rapid in its progress, and the eruptions dried up so speedily, that one cannot doubt that, although the susceptibility of the small-pox was not entirely destroyed, yet it was so partially destroyed as to produce an extremely mitigated small-pox, in respect to both symptoms and eruption, agreeably to what commonly occurs in cases of failure of cow-pock.

4thly. As these cases of failure occurred at the same time, although inoculated in the years 1801, 1804, 1805, it seems to preclude the supposition of any thing like unsusceptibility for a determinate number of years.

5thly. The vaccination being under the direction of two different competent surgeons, the matter being from three different sources, and each time at the most efficacious period, precludes any reason for imputing the failure to the kind of matter or the mode of conducting the practice.

6thly. The scars upon the arms are at least collateral proofs of the affection having been duly produced.

7thly. It does not appear that the children had the subsequent small-pox mitigated in any proportion to the degree of affection by vaccination.

8thly. The circumstance of the successive seizure of four patients in the same family, decidedly prove the disease to be small-pox, if any doubt had arisen on account of the characteristic symptoms.

9thly. Matter has been taken for inoculation from the child George, for further, though unnecessary, proof.

10thly. It seems extremely probable, that, but for previous vaccination, not only John, but that Mary and Lucy, would have had the disease much more severely.

Dr. Pearson has to remark, in addition to the above conclusions,

1st. That the mother of these children had the small-pox, when five years old, very severely; that their father's sister had them still more severely, with what is called the purples; but her father had them slightly.

2dly. As the four children in this family all fell ill within our days of one another, viz. from Saturday to Tuesday, they could not have communicated the disease to one another, but in all probability derived it from one common origin, viz. that of some child ill of the small-pox frequenting the shop, as already said, and conveyed from the mother, who had the care of the shop, to her children, for they had never been in contact with those ill of the small-pox by living or playing with them.

3dly. It is worthy of remark, that, however severely two of these children suffered from the small-pox after cow-pock, it is probable they would have suffered much more, and even have lost their lives, if they had not been previously vaccinated, from the consideration of the constitution of the family on the mother's side. The death of the child, as already said, and the narrow escape with life, (but deformed,) of the eldest daughter, Charlotte, as already mentioned.

COLLECTANEA

